# Successful Prophylactic Endovascular Therapy for a Rapidly Expanding Hepatic Arterial Aneurysm in a Patient with Vascular Ehlers–Danlos Syndrome

**DOI:** 10.3400/avd.cr.20-00144

**Published:** 2021-06-25

**Authors:** Yukihiro Watanabe, Koichi Akutsu, Daisuke Yasui, Fumie Sugihara, Hideki Miyachi, Hiroshi Hayashi, Eiichiro Oka, Hidenori Komiyama, Shin-ichiro Kumita, Wataru Shimizu

**Affiliations:** 1 Department of Cardiovascular Medicine, Nippon Medical School, Tokyo, Japan; 2 Department of Radiology, Nippon Medical School, Tokyo, Japan

**Keywords:** Ehlers–Danlos syndrome type IV, prophylactic endovascular therapy, rapid expansion of arterial aneurysm

## Abstract

Vascular Ehlers–Danlos syndrome (vEDS) causes fatal vascular complications due to vascular fragility. However, invasive therapeutic procedures are generally avoided except in emergencies. We report a case of vEDS presenting with rapid expansion of a hepatic arterial aneurysm successfully treated using prophylactic endovascular therapy. A 43-year-old woman with vEDS confirmed by genetic testing was hospitalized for a symptomatic hepatic arterial aneurysm that expanded rapidly within a week. Prophylactic coil embolization was then successfully performed. Although the general applicability of this approach cannot be determined, prophylactic endovascular therapy can clearly be an option for arterial aneurysms at high risk of rupture.

## Introduction

Ehlers–Danlos syndrome (EDS) refers to a group of rare genetic connective tissue disorders classified into 13 subtypes according to international guidelines. Vascular EDS (vEDS) is a particular type of EDS that is characterized by vascular and visceral complications such as arterial aneurysms; arterial dissection; and the rupture of arteries, the uterus, and gastrointestinal tract. Arterial rupture is the most common cause of death in vEDS.^1)^ Invasive therapeutic procedures such as open surgery and endovascular therapy for vascular complications, in general, are avoided as much as possible except in emergency cases.

Here, we report a case of vEDS with rapid expansion of a hepatic arterial aneurysm that was successfully treated using prophylactic endovascular therapy.

## Case Report

**Figure 1:**
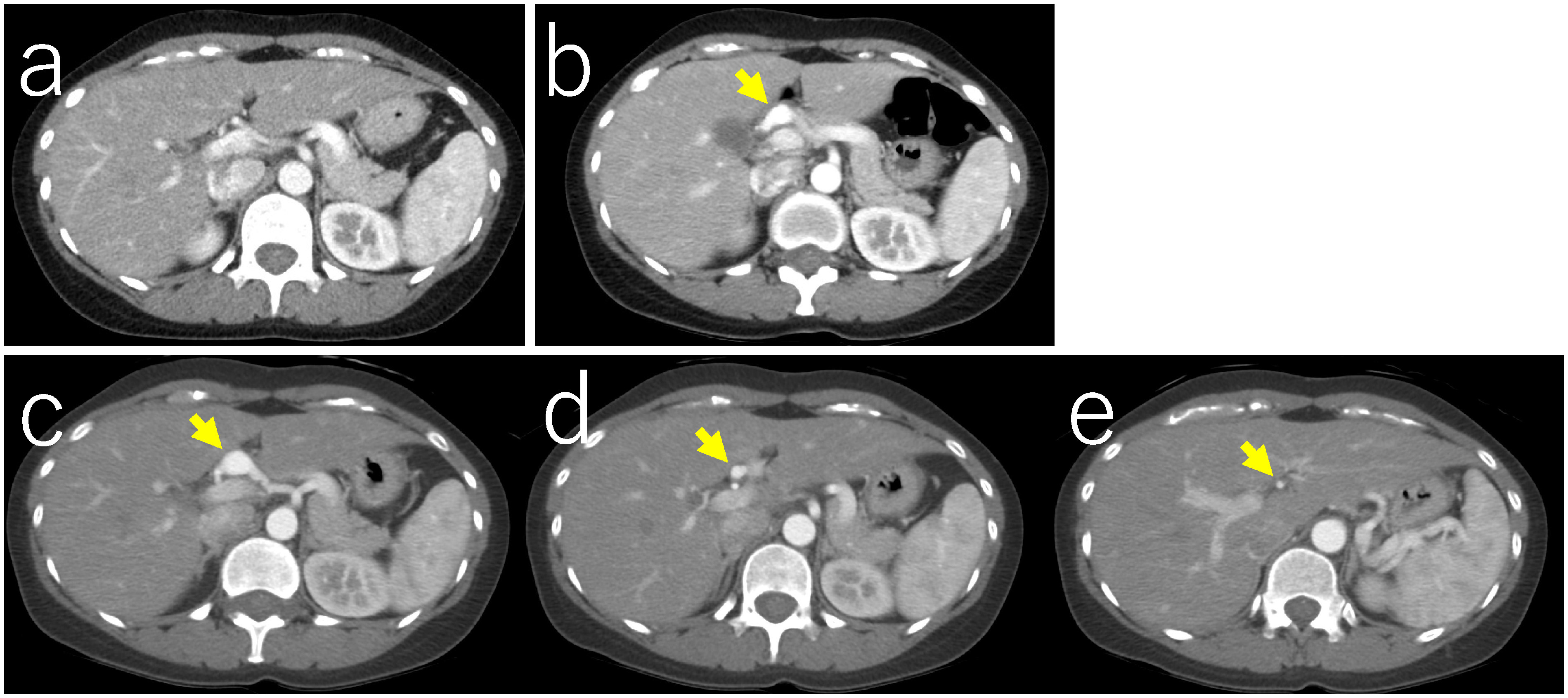
Fig. 1 Contrast-enhanced computed tomography (CT) images showed the rapid expansion of arterial aneurysms. (**a**) At a regular consultation 11 days before the patient’s admission, regular CT once a year at our hospital showed no aneurysm. (**b**) CT image on admission demonstrating a common hepatic arterial aneurysm (7 mm in diameter) (arrow). (**c–e**) Follow-up CT image on day 7 of the patient’s hospitalization showing an expansion of a common hepatic arterial aneurysm (14 mm in diameter), a new proper hepatic arterial aneurysm, and new left hepatic arterial aneurysms (arrow).

A 43-year-old woman was transferred to our hospital complaining of acute abdominal pain. She had a medical history of carotid–cavernous fistula and external iliac artery stenosis and had received endovascular therapy at the age of 39 years at another hospital. Because she had thin, translucent skin as well as a long history of being bruised easily, vEDS was suspected. This was confirmed using genetic testing when the patient was 41 years of age, which revealed a mutation in *COL3A1*. At a regular consultation 11 days before the patient’s admission, computed tomography (CT) imaging (undertaken once a year at our hospital) had shown no signs of aneurysm (**Fig. 1a**), and the patient had not complained of any symptoms.

At presentation, her vital signs were as follows: blood pressure of 122/66 mmHg, heart rate of 87 beats per minute, and body temperature of 37.0°C. The physical examination was unremarkable. Laboratory results revealed a white blood cell count of 10,400/µL, C-reactive protein of 6.4 mg/dL, and D-dimer of 1.5 µg/mL. Her remaining laboratory results were near normal. Contrast-enhanced CT demonstrated a common hepatic arterial aneurysm (7 mm in diameter) that was not present in the previous CT scan (**Fig. 1b**).

The patient was hospitalized for careful observation. We performed contrast-enhanced chest CT and magnetic resonance imaging of the head to check for vascular complications in the whole body, but no other vascular lesions were identified. The patient did not complain of abdominal pain again after admission. However, follow-up CT on day 7 of her hospitalization showed a rapid expansion of a common hepatic arterial aneurysm (14 mm in diameter), a new proper hepatic arterial aneurysm, and new left hepatic arterial aneurysms (**Figs. 1c–1e**, respectively). As it was feared that this rapid expansion would cause arterial rupture, we considered performing prophylactic therapeutic procedures such as open surgery or endovascular therapy before arterial rupture could occur. However, in general, invasive therapeutic procedures in patients with vEDS should be avoided as much as possible because they may lead to iatrogenic complications caused by extremely fragile arteries. Nonetheless, after careful discussion with vascular surgeons and radiologists, we decided to attempt prophylactic endovascular therapy to avoid arterial rupture.

**Figure 2:**
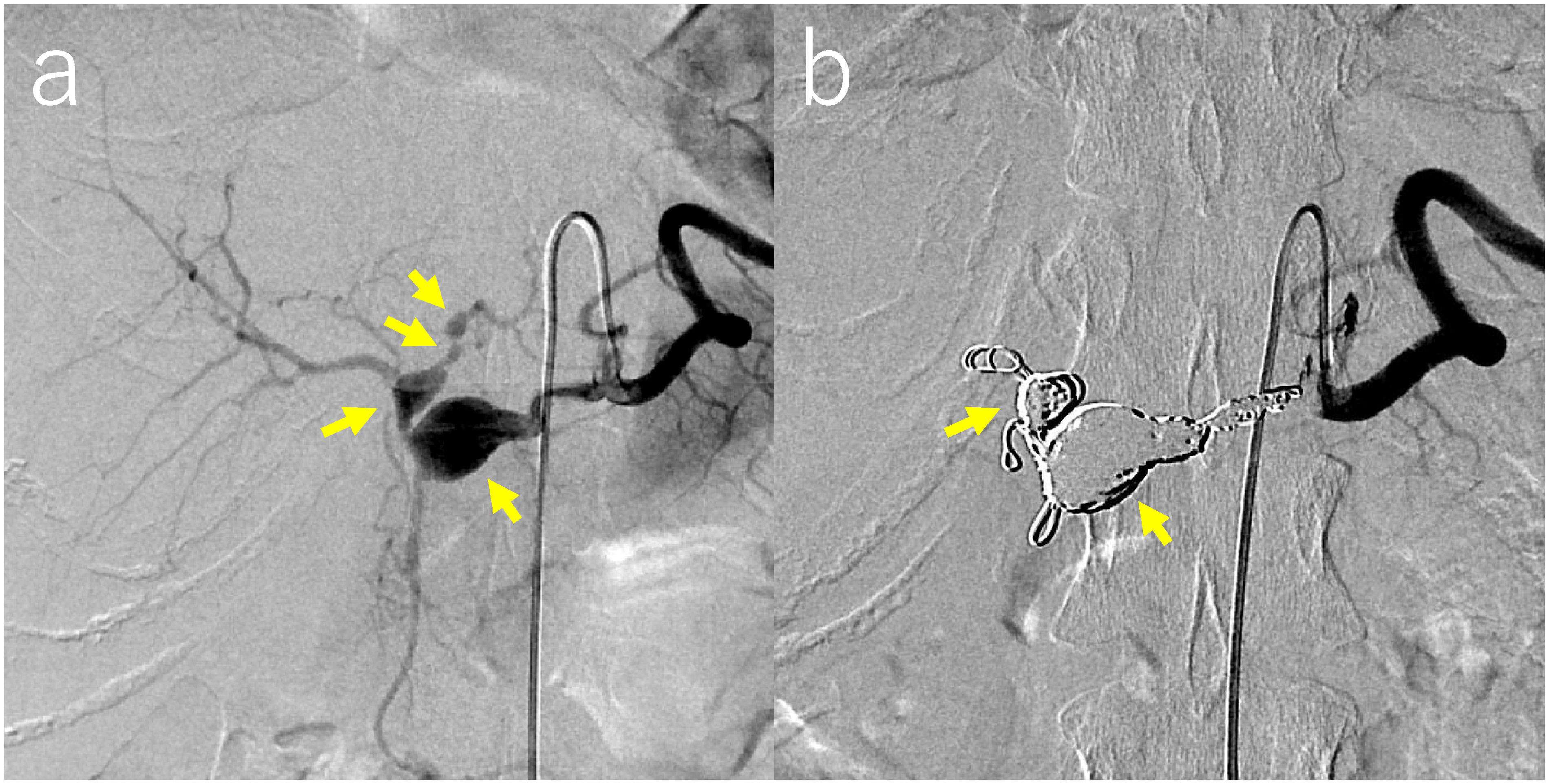
Fig. 2 Angiography showing therapeutic procedures. (**a**) A celiac artery angiogram revealed a proper and common hepatic arterial aneurysm and left hepatic arterial aneurysms (arrows). (**b**) Coil embolization was successfully performed, and the aneurysms were completely occluded after coil embolization (arrows).

Ultrasonically guided percutaneous puncture of the anterior wall of the left common femoral artery was performed to insert a 4-F sheath (Medikit, Tokyo, Japan) under local anesthesia. A celiac artery angiogram with a 4-F diagnostic catheter (Medikit, Tokyo, Japan) revealed a proper and common hepatic arterial aneurysm and left hepatic arterial aneurysms (**Fig. 2a**). A microcatheter (Coiling Support®, HI-LEX Corporation, Takarazuka, Japan) was selectively inserted into the proper hepatic arterial aneurysm. Detachable coils (Target®, Stryker, Kalamazoo, MI, USA) were used for packing because these are soft and their 3D conformation is suitable for tight packing. Framing was performed using XXL 360 coils (primary coil diameter: 0.017 inches), followed by filling using XL soft coils (primary coil diameter: 0.014 inches). The volume of the proper and common hepatic artery aneurysm was calculated and added before the procedure, using a region of interest volume calculation algorithm of a dedicated software (OsiriX MD®, Pixmeo Sarl, Bernex, Switzerland). The total volume was 2.25 cm^3^. The packing density was monitored during the embolization. Nine 0.017-inch microcoils (total length: 370 cm) were deployed in the aneurysms, and the final packing density was 24.1%. After packing the aneurysm, a microcatheter was drawn back to the common hepatic artery, and embolization was subsequently performed. Aneurysms were completely occluded using 19 coils (**Fig. 2b**). Manual compression of the puncture site was performed for 10 min and complete hemostasis was obtained, confirmed using ultrasonography. The patient was required to rest in bed for 6 h following the procedure.

The patient’s postoperative course was uneventful. Laboratory results yielded normal values with no evidence of bleeding or liver dysfunction. Ultrasound imaging confirmed the lack of puncture site complications. There was no exacerbation of abdominal pain after embolization. Two days after treatment, follow-up CT imaging revealed the development of an extrahepatic collateral pathway—the right subphrenic artery, but with no obvious complications. The patient was discharged from the hospital 7 days after treatment. Follow-up CT imaging performed 3 and 6 months after treatment showed no expansion of the hepatic arterial aneurysm or any vascular lesions. The patient has not experienced any complications in the subsequent 14 months and will continue to receive follow-up examinations regularly.

## Discussion

vEDS syndrome is a rare connective tissue disorder caused by a *COL3A1* mutation. Patients with vEDS experience premature death, with a median life expectancy of 51 years^2)^ due to vascular complications, especially arterial rupture.^1)^ However, invasive therapeutic procedures before fatal vascular complications should generally be avoided as much as possible because they may lead to iatrogenic complications caused by extremely fragile arteries. In reports of invasive therapeutic procedures, the mortality rates of open surgery (30%) and endovascular therapy (24%) have been high.^3)^ Therefore, conservative medical management is often selected for vascular complications, and invasive therapeutic procedures are limited to cases of acute arterial rupture. As a result, it has been reported that 70% of interventional procedures were performed in an emergency or urgently.^4)^

The validity of prophylactic treatment for vascular complications in patients with vEDS is controversial and should be discussed on a case-by-case basis with regard to balancing the risk of arterial rupture and iatrogenic complications during interventional procedures. However, because the risk of rupture is extremely high for rapidly expanding aneurysms or those with a large diameter, prophylactic treatment should be considered. Recently, although the number of cases treated is still small, prophylactic treatment for patients with vEDS has been reported.^4–6)^ One report described 30 interventional procedures in patients with vEDS, of which 30% were prophylactic (9/30). Interventional procedures were performed in 33% (10/30) and 7% (2/30) of cases for large aneurysms and rapid aneurysm expansion, respectively. The prophylactic interventional procedures resulted in good survival rate with no hospital deaths.^4)^ Some other reports also described prophylactic interventional procedures with good outcomes.^5,6)^ Recently reported studies of interventional procedures are shown in **Table 1**. Most elective procedures were performed prophylactically. Thus, there are increasing numbers of reports of prophylactic therapy performed as a possible option for an arterial aneurysm at a high risk of rupture.

**Table 1:** Table 1 A summary of recent studies reporting interventional procedures

Author	Publication year	Urgency/n	Treatment/n	In-hospital mortality, n (%)
Oderich et al.^4)^	2005	Elective/9	Unknown	0
		Emergency/21	Unknown	2 (11)
Brooke et al.^5)^	2010	Elective/10	OS/8	1 (13)
		Emergency/2	EVT/2	0
			OS/1	0
			EVT/1	0
Shalhub et al.^6)^	2014	Elective/21*	Unknown	0
		Emergency/29*	Unknown	3 (10)
Okada et al.^8)^	2014	Emergency/7	EVT/7	1 (14)

*Diagnosis of vEDS was known in 42% cases prior to intervention.OS: open surgery; EVT: endovascular therapy; vEDS: vascular Ehlers–Danlos syndrome

Comparing prophylactic open surgery and endovascular therapy based on previous reports, it has not been determined which treatment is superior. The number of cases treated prophylactically has been small and the results variable. Deciding which treatment is suitable for a patient should be done on a case-by-case basis, considering the pathology and general condition of that individual. For the present case, we selected endovascular therapy because it was appropriate for pathological indications and less invasive. Stentgraft implantation or embolization was considered treatment options. Hepatic arterial flow can be preserved using stentgraft implantation, but there is a considerable risk of vascular injury because the insertion of a large-bore guiding catheter (6-F inner diameter) is required for stent delivery. There was also a risk of vascular injury caused by the edge of the stentgraft. Moreover, the diameter of the distal landing zone (the left and right hepatic arteries) was <3 mm and stent infolding was thought likely to occur, possibly leading to occlusion. Thus, embolization of the aneurysm was selected as the best available option. Isolation was considered at first; however, cannulation to multiple-branch vessels would have been required for this procedure. It is known that even a minimal vascular insult may induce remote vascular catastrophe in vEDS,^7)^ and therefore, cannulation should be avoided as much as possible. Thus, we planned to undertake packing of the aneurysm. The aim of the procedure was to balance the merit of avoiding lethal bleeding against the risk of procedure-related complications. Extensive filling may lead to the rupture of the aneurysm during the procedure, so we set 20% as the target filling rate. Moreover, packing of only the proper and common hepatic arterial aneurysms was planned, because hemorrhage inside the hilar plate can be managed conservatively.

There are several options regarding the selection of embolization material. Coils have been employed in most of the reported cases in the small number of currently available publications.^8–10)^ In some reported cases,^8,9)^ N-butyl 2-cyanoacrylate (NBCA) was used along with coils. However, microcatheter adhesion can occur, and therefore, indications for their use should be thoroughly considered before this procedure. In the present case, we decided not to use NBCA because significant packing of the aneurysm was obtained using only microcoils. Intraprocedural rupture and iatrogenic intimal injury occurred in 43% of these types of procedures (3/7) in one published article.^8)^ Differences in risks for complications between different embolization materials are not clearly described in previous reports. Thus, operators should carefully select the appropriate embolization method on a case-by-case basis.

A diagnostic confirmation of vEDS before invasive therapeutic procedures is important for a successful outcome. Operators will perform therapeutic procedures with maximum care if they know the patient has a definite diagnosis of vEDS before treatment. This is illustrated in a report of 50 invasive procedures in patients with vEDS including open surgery and endovascular therapy. It was reported that fewer intraoperative deaths (0% vs. 14%; p=0.036) and postoperative complications (14% vs. 62%; p<0.001) occurred in patients with a known diagnosis of vEDS before therapeutic procedures compared with those without such a diagnosis. These results might be biased because patients without a known vEDS diagnosis before therapeutic procedure were more likely to receive emergency treatment (81% vs. 41%; p=0.005) and undergo open surgery (81% vs. 48%; p=0.019). However, in elective procedures (21/50), there were fewer postoperative complications (5% vs. 55%; p<0.001) without in-hospital deaths.^6)^ Thus, preoperative diagnosis of vEDS and elective rather than emergency procedures lead to more successful results. In the case of endovascular therapy for vEDS, unnecessary angiography should be avoided because the injection of contrast material may itself cause arterial injuries; the use of liquid embolic materials and soft coils is recommended for coil embolization to protect fragile arteries.^8)^ Generally, using a small caliber sheath (4-F or 5-F) and a single wall puncture with ultrasound guidance is expected to decrease puncture site complications. In the present case, a diagnosis of vEDS was confirmed using genetic analysis before endovascular therapy and all protective endovascular procedures were performed with maximum caution to protect fragile arteries; for example, in principle, collateral flow from the superior mesenteric, left gastric and right subphrenic arteries should also be evaluated after embolization. However, cannulation to these vessels comes with added risks of vascular injury and so was not performed in this case. This was likely to have been one of the important conditions responsible for the successful outcome from our endovascular therapy in this patient.

## Conclusion

In patients with vEDS, prophylactic endovascular therapy can be an option to avoid arterial rupture in the case of an arterial aneurysm at high risk of rupture. After careful evaluation of the risks and benefits, elective prophylactic endovascular therapy of a rapidly expanding arterial aneurysm before arterial rupture should be considered.

## Additional Note

Written informed consent was obtained from the patient for publication of this case report and accompanying images.
